# 597. False Positive Carbapenemase Test in Carbapenem Resistant *Acinetobacter baumannii*

**DOI:** 10.1093/ofid/ofad500.664

**Published:** 2023-11-27

**Authors:** Lili Tao, Emily Bentley, Carmella Russo, Ayesha Khan, Daniel D Rhoads, Romney Humphries

**Affiliations:** Vanderbilt University Medical Center, Nashville, Tennessee; Vanderbilt University Medical Center, Nashville, Tennessee; Vanderbilt University Medical Center, Nashville, Tennessee; Vanderbilt University Medical Center, Nashville, Tennessee; Cleveland Clinic Foundation, Cleveland, OH; Vanderbilt University Medical Center, Nashville, Tennessee

## Abstract

**Background:**

The detection of carbapenemases in carbapenem resistance gram negative organisms is necessary for appropriate antibiotic treatment and infection prevention. Lateral flow testing for the most common carbapenemases, KPC, NDM, VIM, IMP and OXA-48 is FDA-cleared for use in *Enterobacterales* and *Pseudomonas aeruginosa*. We evaluated use this assay for the detection of carbapenemases in carbapenem-resistant *Acinetobacter baumannii* (CRAB), which yielded false positives for the IMP carbapenemase.

**Methods:**

CRAB (n=22) isolated from routine clinical cultures at two sites were evaluated. Isolates were tested by the NG-Test CARBA 5 (NG Biotech, Guipry, France). Phenotypic metallo-β-lactamase testing was performed using meropenem-EDTA disk and imipenem-EDTA disk method, including positive controls (NCTC 13476, *Escherichia coli*, IMP+; BAA 2146, *Klebsiella pneumoniae*, NDM+) and negative controls (BAA 1706, *K. pneumoniae*, negative for carbapenemase, BAA 1705, *K. pneumoniae*, KPC+). The absence of *bla*_IMP_ was further confirmed by whole genome sequencing (WGS) using MiSeq (Illumina, CA). The identification of antibiotic resistance genes using WGS data was performed using BV-BRC pipelines (https://www.bv-brc.org).

**Results:**

Of 22 isolates, 14 (66.7%) tested positive for IMP using NG-Test CARBA 5. No other carbapenemases were detected in these isolates by the lateral flow assay. Metallo-β-lactamase activity was not detected in any of the CRAB isolates. WGS and antibiotic resistance gene identification using Comprehensive Antibiotic Resistance Database (CARD) and National Database of Antibiotic Resistant Organisms failed to identify any *bla*_IMP_ or other metallo-β-lactamase genes in the CRAB isolates. Β-lactamase genes detected by WGS in CRAB isolates were listed in Table 1.

**Figure:**
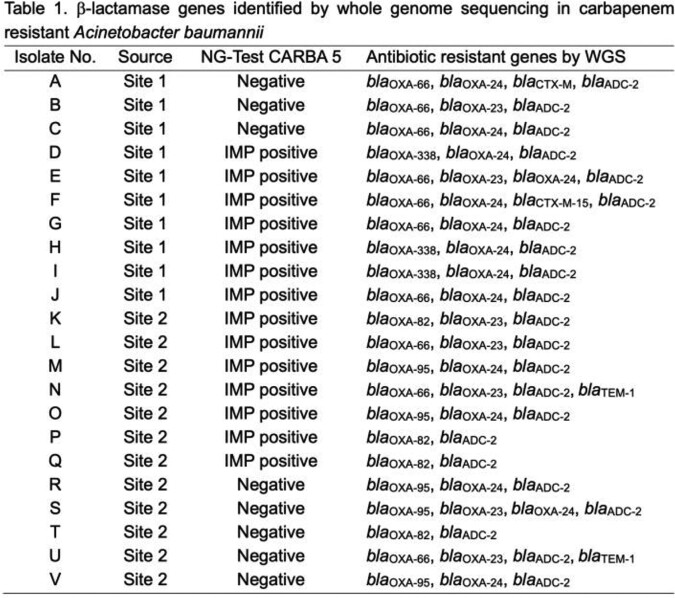

**Conclusion:**

Positive IMPs detected by NG-Test CARBA 5 in CRAB isolates were most likely false positive results. Further confirmative tests may be indicated for the off-label use of NG-Test CARBA 5.

**Disclosures:**

**Daniel D. Rhoads, MD**, Hardy Diagnostics: Grant/Research Support **Romney Humphries, PhD, D(ABMM), M(ASCP)**, Melinta: Advisor/Consultant|Merck: Advisor/Consultant|Shionogi: Advisor/Consultant|Ventorx: Advisor/Consultant

